# Trends in the Burden of Chronic Liver Disease Among Hospitalized US Adults

**DOI:** 10.1001/jamanetworkopen.2020.1997

**Published:** 2020-04-02

**Authors:** Grishma Hirode, Sammy Saab, Robert J. Wong

**Affiliations:** 1 Division of Gastroenterology and Hepatology, Alameda Health System, Highland Hospital, Oakland, California; 2 David Geffen School of Medicine, Department of Medicine, University of California at Los Angeles

## Abstract

**Importance:**

One factor associated with the rapidly increasing clinical and economic burden of chronic liver disease (CLD) is inpatient health care utilization.

**Objective:**

To understand trends in the hospitalization burden of CLD in the US.

**Design, Setting, and Participants:**

This cross-sectional study of hospitalized adults in the US used data from the National Inpatient Sample from 2012 to 2016 on adult CLD-related hospitalizations. Data were analyzed from June to October 2019.

**Main Outcomes and Measures:**

Hospitalizations identified using a comprehensive review of CLD-specific *International Classification of Diseases, Ninth Revision, Clinical Modification *and *International Statistical Classification of Diseases, Tenth Revision, Clinical Modification* codes. Survey-weighted annual trends in national estimates of CLD-related hospitalizations, in-hospital mortality, and hospitalization costs, stratified by demographic and clinical characteristics.

**Results:**

This study included 1 016 743 CLD-related hospitalizations (mean [SD] patient age, 57.4 [14.4] years; 582 197 [57.3%] male; 633 082 [62.3%] white). From 2012 to 2016, the rate of CLD-related hospitalizations per 100 000 hospitalizations increased from 3056 (95% CI, 3042-3069) to 3757 (95% CI, 3742-3772), and total inpatient hospitalization costs increased from $14.9 billion (95% CI, $13.9 billion to $15.9 billion) to $18.8 billion (95% CI, $17.6 billion to $20.0 billion). Mean (SD) patient age increased (56.8 [14.2] years in 2012 to 57.8 [14.6] years in 2016) and, subsequently, the proportion with Medicare also increased (41.7% [95% CI, 41.1%-42.2%] to 43.6% [95% CI, 43.1%-44.1%]) (*P* for trend < .001 for both). The proportion of hospitalizations of patients with hepatitis C virus was similar throughout the period of study (31.6% [95% CI, 31.3%-31.9%]), and the proportion with alcoholic cirrhosis and nonalcoholic fatty liver disease showed increases. The mortality rate was higher among hospitalizations with alcoholic cirrhosis (11.9% [95% CI, 11.7%-12.0%]) compared with other etiologies. Presence of hepatocellular carcinoma was also associated with a high mortality rate (9.8% [95% CI, 9.5%-10.1%]). Cost burden increased across all etiologies, with a higher total cost burden among hospitalizations with alcoholic cirrhosis ($22.7 billion [95% CI, $22.1 billion to $23.2 billion]) or hepatitis C virus ($22.6 billion [95% CI, $22.1 billion to $23.2 billion]). Presence of cirrhosis, complications of cirrhosis, and comorbidities added to the CLD burden.

**Conclusions and Relevance:**

Over the study period, the total estimated national hospitalization costs in patients with CLD reached $81.1 billion. The inpatient CLD burden in the US is likely increasing because of an aging CLD population with increases in concomitant comorbid conditions.

## Key Points

**Question:**

Is the burden of chronic liver disease–related hospitalization on the US health care system changing over time?

**Findings:**

In this cross-sectional study of 1 016 743 adult hospitalizations related to chronic liver disease, the inpatient burden increased in association with an aging population with increasing comorbidities. While inpatient burden attributable to hepatitis C virus remained significant, the burden due to alcoholic cirrhosis and nonalcoholic fatty liver disease also increased.

**Meaning:**

The increasing burden of hospitalizations related to chronic liver disease emphasizes this disease’s clinical and economic impact and the importance of optimizing outpatient management to prevent complications leading to the need for hospitalization.

## Teaser

This cross-sectional study assesses trends in the burden of chronic liver disease among hospitalized patients on the US health care system.

## Introduction

The landscape of chronic liver disease (CLD) in the US is rapidly changing. This condition is currently the fourth leading cause of death among persons aged 45 to 64 years.^1^ With improvements in the management and treatment of viral hepatitis, the burden of liver disease is shifting toward alcoholic liver disease (ALD) and nonalcoholic fatty liver disease (NAFLD).^2,3,4,5^ A recent US population-based study focusing on cirrhosis-related mortality observed that increasing cirrhosis death rates in the US were largely driven by alcoholic cirrhosis, with the greatest increases seen among young adults aged 25 to 34 years.^6^

Inpatient hospitalization and care are a major contributor to health care resource utilization among patients with CLD.^7,8,9,10^ Few prior studies have used large-scale population-based data sets to evaluate time trends in the total CLD burden and among individual CLD etiologies,^4,11,12^ cirrhosis, and complications associated with cirrhosis.^13^ Asrani et al^14,15^ illustrated trends in the increasing burden of CLD using patient data from hospitalizations in a large and diverse metroplex. Another study^4^ used the National Health and Nutrition Examination Survey database to provide a more generalizable, comprehensive overview of CLD epidemiology in the US. While the inpatient burden remains unknown, these studies highlight the significant clinical impact of CLD. A more thorough understanding of trends in the hospitalization burden of CLD is an important first step in identifying potential areas of focus for public health initiatives and quality improvement programs to ameliorate CLD-related health care resource utilization in the US.

This study specifically aims to comprehensively evaluate the inpatient clinical and economic burden of CLD using a nationally representative sample of CLD-related hospitalizations and further analyze demographic and etiology-specific differences.

## Methods

This study used deidentified data and was granted exempt status by the Alameda Health System institutional review board. We conducted analysis of these data from June 2019 to October 2019. This study followed the Strengthening the Reporting of Observational Studies in Epidemiology (STROBE) reporting guideline.^16^

### Data Source

This study evaluated 2012 to 2016 data from the National Inpatient Sample (NIS).^17^ The NIS is the largest all-payer inpatient database of hospital discharges in the US and is maintained as part of the Healthcare Cost and Utilization Project by the Agency for Healthcare Research and Quality. The NIS contains deidentified information regarding each hospitalization, including patient demographic characteristics, admission status, discharge diagnoses, procedures, comorbid conditions, outcomes, and hospital charges. If race and ethnicity were provided in separate data elements, ethnicity took precedence over race in setting the Healthcare Cost and Utilization Project value for race. Participating hospitals are sampled based on characteristics such as size, location (rural vs urban), geographic region, ownership, and teaching status. Starting in 2012, the NIS modified its method of data acquisition to include a systematic sampling of 20% of discharges from all hospitals stratified by hospital, US Census division, ownership status, urban vs rural location, teaching status, and bed size, as well as patient diagnosis-related group and admission month.^17^ In 2015, data elements and data structure for the NIS were changed again.^18^ Trends based on diagnoses or procedures were affected because, starting October 2015, hospital administrative data in the US began using *International Classification of Diseases, Tenth Revision, Clinical Modification/Procedure Coding System (ICD-10-CM/PCS)*. In the 2015 NIS, the first 9 months contain *International Classification of Diseases, Ninth Revision, Clinical Modification (ICD-9-CM)* codes and the last 3 months contain *ICD-10-CM/PCS* codes.^19,20,21^ The Elixhauser Comorbidity Index software indicators were not available beginning October 1, 2015, because the *ICD-10-CM* version of the software is still under development.^18^

### Study Population and Variables

The sample included CLD-related hospitalizations of US adults (aged ≥18 years) between January 1, 2012, and December 31, 2016. We defined CLD using a comprehensive review of *ICD-9-CM* codes based on prior published algorithms^14,15^ from January 1, 2012, to September 30, 2015, and corresponding *ICD-10-CM* codes starting October 1, 2015 (eFigure and eTable 1 in the Supplement). To minimize ascertainment bias, we classified a hospitalization as CLD related if it was associated with (1) a primary discharge diagnosis of CLD or (2) a primary discharge diagnosis of another liver-related cause (eg, cirrhosis) in combination with a secondary diagnosis of CLD (eTable 1 in the Supplement).

Among CLD-related hospitalizations, we stratified hospitalizations by 4 main etiologies (alcoholic cirrhosis, hepatitis B virus [HBV] infection, hepatitis C virus [HCV] infection, and NAFLD), as these etiologies combined account for most of the burden of CLD among hospitalized patients in the US. Adult hospitalizations with alcoholic cirrhosis were identified by first isolating all hospitalizations with cirrhosis listed as a diagnosis (eTable 2 in the Supplement). From the cohort with cirrhosis, we excluded patients with autoimmune hepatitis, viral hepatitis, hemochromatosis, only NAFLD-specific codes (*ICD-9:* 571.5, 571.8-9; *ICD-10:* K75.81), primary biliary cholangitis, and primary sclerosing cholangitis listed as a diagnosis (eTable 2 in the Supplement). We then defined alcoholic cirrhosis as hospitalizations with ALD or alcohol use disorder in addition to cirrhosis^22^ (eTable 2 in the Supplement). Adult hospitalizations with NAFLD were identified by first using *ICD* codes for liver disease without mention of alcohol (*ICD-9:* 571.5, 571.8-9, 567.23, 572.2-4, 456.0-2x, 789.5x; *ICD-10:* I85.xx, K65.2, K72.1x, K72.9x, K74.0-2, K74.6x, K75.8x, K75.9, K76.0, K76.6-9, K77, R18.x) and then excluding patients with chronic liver disease with mention of alcohol or alcohol use disorder (eTable 2 in the Supplement), alcoholic cirrhosis, and other etiologies of acute or chronic liver disease^8,11,23^ (autoimmune hepatitis, Budd-Chiari syndrome, chronic passive congestion of liver, clonorchiasis, disorders of porphyrin and bilirubin metabolism, echinococcus of liver, fascioliasis, Gaucher disease, hemochromatosis, viral hepatitis, lysosomal acid lipase deficiency and other lipoid disorders, opisthorchiasis, other amyloidosis, other deficiencies of circulating enzymes, other disorders of the liver, primary biliary cholangitis, primary sclerosing cholangitis, syphilis of the liver, or Wilson disease) (eTable 2 in the Supplement). Patients with alcohol abuse listed as an Elixhauser comorbidity were included in the alcoholic cirrhosis cohort and excluded from the NAFLD cohort for data using *ICD-9-CM* codes.

We additionally evaluated for the presence of hepatocellular carcinoma, cirrhosis, and cirrhosis-related complications, including ascites, esophageal varices, hepatic encephalopathy, and hepatorenal syndrome (eTable 1 and eTable 2 in the Supplement).

### Outcome Measures

We estimated the number of CLD-related hospitalizations in each study year. We further described demographic (age, sex, race, and insurance), clinical (liver disease etiology; hepatocellular carcinoma; cirrhosis and concurrent presence of 0, 1, or ≥2 cirrhosis-related complications among patients with cirrhosis; and comorbidities), and hospital characteristics (region, bed size, location, and teaching status) of the sample in each study year. Among CLD-related hospitalizations, we examined year-specific in-hospital mortality and hospitalization costs, stratified by demographic and clinical characteristics.

Hospitalization charges as provided within the data set were inflation adjusted to and reported as 2016 US dollars using the Consumer Price Index maintained by the US Department of Labor.^24^ Hospitalization charges may be confounded by payer policies and other factors unrelated to resource utilization. Therefore, hospitalization costs were calculated for each hospitalization using hospital-specific cost to charge ratios developed by the Agency for Healthcare Research and Quality.^25^

### Missing Data

Hospitalizations with missing data on age and/or sex were excluded. Hospitalizations with missing data on race (4.4%) were coded with a dummy variable to represent the missing data. In all, 0.2% of observations were missing data on insurance status, 0.07% of hospitalizations were missing data on in-hospital mortality, and 2.0% were missing data on costs. Because missingness for each variable with missing observations was 5% or less, and because the study was a cross-sectional analysis of trends, observations with missing data were excluded from the analysis of the respective variable.^27,28^

### Statistical Analysis

Data were survey-weighted using individual discharge weights to obtain descriptive statistics and national estimates. We analyzed time trends in the rate of CLD-related hospitalizations, and among CLD-related hospitalizations, we analyzed trends in the prevalence of demographic and clinical characteristics. We further analyzed trends in in-hospital mortality, mean hospitalization costs, and total hospitalization costs; we performed subgroup analyses by demographic and clinical characteristics. Secular trends were assessed after regressing all outcomes on year, modeled as a continuous variable. We used logistic regression for demographic and clinical outcomes and in-hospital mortality and linear regression after log-transforming the outcome for mean hospitalization costs. We used Poisson regression with count variables to test for trend in the rate of CLD-related hospitalizations. Between-group comparisons used Pearson χ^2^ tests for binary and categorical variables.

A 2-tailed *P* < .05 was considered statistically significant. Statistical analyses were performed using Stata version 14.0 (StataCorp). We followed the research practices recommended when using NIS data.^26^

## Results

### Characteristics of the Study Cohort

Among 1 016 743 CLD-related hospitalizations from 2012 to 2016, the mean (SD) patient age was 57.4 (14.4) years; 582 197 (57.3%) were male; and 633 082 (62.3%) were white, 138 068 (13.6%) were African American, and 134 662 (13.2%) were Hispanic (Table 1). The largest proportion of hospitalizations were for patients aged 45 to 64 years (53.0% [95% CI, 52.8%-53.1%]). Medicare was the most common insurance type (42.7% [95% CI, 42.5%-42.9%]), followed by Medicaid or private insurance. The mean (SD) length of hospitalization was 6.3 (8.0) days. The most common liver disease etiology was HCV (31.6% [95% CI, 31.3%-31.9%]). Overall, 538 720 hospitalizations (95% CI, 53.0% [52.8%-53.2%]) had cirrhosis, and 25.7% (95% CI, 25.5%-25.8%) had cirrhosis with 1 cirrhosis-related complication present. Hypertension and diabetes were the most prevalent comorbidities (Table 1).

**Table 1.** Trends in the Clinical and Demographic Characteristics of the Study Cohort

Characteristic	Hospitalizations related to chronic liver disease, weighted % (95% CI)						*P* value for trend
	Total (n = 1 016 743)	2012 (n = 187 690)	2013 (n = 192 614)	2014 (n = 201 069)	2015 (n = 208 656)	2016 (n = 226 714)	
Chronic liver disease–related hospitalizations per 100 000, No.	3372	3056	3214	3380	3459	3757	<.001
Age, mean (SD), y	57.4 (14.4)	56.8 (14.2)	57.1 (14.3)	57.3 (14.4)	57.7 (14.5)	57.8 (14.6)	<.001
Age, y							
<25	1.5 (1.5-1.6)	1.6 (1.5-1.6)	1.6 (1.6-1.7)	1.6 (1.5-1.6)	1.5 (1.5-1.6)	1.5 (1.4-1.5)	.02
25-44	15.7 (15.6-15.8)	15.6 (15.3-15.9)	15.4 (15.1-15.7)	15.6 (15.4-15.9)	15.7 (15.4-16.0)	16.1 (15.8-16.4)	.01
45-64	53.0 (52.8-53.1)	55.5 (55.1-55.9)	54.4 (54.0-54.8)	53.4 (53.0-53.8)	51.9 (51.5-52.2)	50.3 (49.9-50.6)	<.001
≥65	29.8 (29.6-30.0)	27.4 (26.9-27.9)	28.6 (28.2-29.2)	29.4 (28.9-29.9)	30.9 (30.4-31.4)	32.2 (31.7-32.6)	<.001
Sex							
Male	57.3 (57.1-57.4)	57.8 (57.5-58.1)	57.5 (57.2-57.8)	57.2 (56.9-57.7)	56.9 (56.6-57.2)	57.0 (56.7-57.3)	<.001
Female	42.7 (42.6-42.9)	42.2 (41.9-42.5)	42.5 (42.2-42.8)	42.8 (42.5-43.1)	43.1 (42.8-43.4)	43.0 (42.7-43.3)	<.001
Race							
White	62.3 (61.7-62.8)	61.6 (60.2-63.0)	62.1 (60.7-63.5)	62.6 (61.2-64.0)	62.4 (61.1-63.7)	62.5 (61.2-63.8)	.35
African American	13.6 (13.3-13.9)	14.1 (13.3-15.0)	13.6 (12.8-14.4)	13.4 (12.7-14.2)	13.4 (12.7-14.2)	13.5 (12.8-14.2)	.26
Hispanic	13.2 (12.9-13.6)	13.0 (12.0-14.0)	13.1 (12.2-14.2)	13.0 (12.1-14.0)	13.5 (12.5-14.4)	13.6 (12.7-14.6)	.31
Asian/Pacific Islander	2.5 (2.4-2.6)	2.3 (2.1-2.6)	2.5 (2.2-2.8)	2.5 (2.2-2.8)	2.6 (2.4-2.9)	2.6 (2.3-2.8)	.18
Other	4.1 (3.9-4.3)	4.6 (3.9-5.3)	3.8 (3.4-4.3)	4.1 (3.7-4.5)	3.8 (3.4-4.2)	4.0 (3.6-4.5)	.26
Missing	4.4 (4.0-4.7)	4.4 (3.7-5.4)	4.9 (4.0-5.9)	4.5 (3.6-5.5)	4.3 (3.5-5.2)	3.8 (3.1-4.8)	.17
Insurance							
Medicare	42.7 (42.5-42.9)	41.7 (41.1-42.2)	42.3 (41.8-42.8)	42.7 (42.2-43.3)	43.1 (42.6-43.6)	43.6 (43.1-44.1)	<.001
Medicaid	23.5 (23.2-23.8)	21.6 (20.9-22.2)	21.3 (20.7-22.0)	24.5 (23.7-25.3)	24.6 (23.9-25.4)	24.9 (24.2-25.7)	<.001
Private, including health maintenance organization	22.4 (22.1-22.6)	22.4 (21.8-23.0)	22.0 (21.4-22.6)	22.5 (21.9-23.2)	22.7 (22.1-23.3)	22.1 (21.6-22.7)	.86
Self-pay	7.1 (6.9-7.3)	9.1 (8.6-9.5)	8.8 (8.4-9.3)	6.5 (6.1-6.9)	5.8 (5.4-6.2)	5.7 (5.3-6.1)	<.001
No charge	0.8 (0.7-0.8)	0.8 (0.7-1.0)	1.2 (1.0-1.4)	0.7 (0.6-0.8)	0.6 (0.5-0.8)	0.6 (0.5-0.7)	<.001
Other	3.6 (3.5-3.8)	4.5 (4.2-4.9)	4.4 (4.0-4.7)	3.1 (2.9-3.4)	3.1 (2.9-3.4)	3.1 (2.9-3.4)	<.001
Hospital region							
Northeast	18.4 (17.8-18.9)	19.3 (17.4-21.4)	18.8 (16.8-20.9)	18.6 (16.7-20.6)	17.6 (15.9-19.6)	17.7 (15.9-19.6)	.20
Midwest	19.2 (18.7-19.8)	19.7 (17.9-21.7)	19.8 (18.0-21.9)	19.0 (17.2-20.9)	18.8 (17.1-20.6)	18.9 (17.2-20.8)	.42
South	39.7 (39.1-40.3)	38.8 (36.3-41.3)	39.3 (36.8-41.8)	39.8 (37.4-42.3)	40.1 (37.7-42.5)	40.2 (37.9-42.6)	.40
West	22.7 (22.2-23.3)	22.2 (20.3-24.2)	22.1 (20.2-24.1)	22.6 (20.7-24.7)	23.5 (21.6-25.6)	23.2 (21.3-25.2)	.33
Hospital bed size							
Small	15.2 (14.8-15.6)	12.5 (11.4-13.8)	12.4 (11.3-13.6)	17.1 (15.8-18.5)	16.5 (15.2-17.8)	17.1 (15.8-18.4)	<.001
Medium	27.6 (27.1-28.1)	26.0 (24.1-27.9)	25.9 (24.0-27.9)	28.4 (26.4-30.4)	29.1 (27.2-31.0)	28.2 (26.3-30.1)	.03
Large	57.2 (56.6-57.8)	61.5 (59.3-63.7)	61.7 (59.4-63.9)	54.6 (52.2-56.9)	54.5 (52.2-56.8)	54.7 (52.4-57.0)	<.001
Hospital location and teaching status							
Rural	8.1 (7.9-8.4)	9.3 (8.4-10.2)	9.3 (8.5-10.3)	7.7 (6.9-8.6)	7.3 (6.6-8.0)	7.2 (6.5-7.9)	<.001
Urban, nonteaching	29.3 (28.8-29.8)	35.9 (33.8-38.0)	35.3 (33.2-37.4)	25.3 (23.5-27.1)	25.9 (24.2-27.7)	25.3 (23.6-27.1)	<.001
Urban, teaching	62.6 (62.1-63.2)	54.9 (52.5-57.2)	55.4 (53.0-57.8)	67.0 (65.0-69.0)	66.8 (64.8-68.7)	67.5 (65.6-69.4)	<.001
Length of stay, mean (SD), d	6.3 (8.0)	6.3 (8.2)	6.2 (8.1)	6.4 (8.3)	6.0 (7.2)	6.4 (8.2)	.77
Alcoholic cirrhosis	24.5 (24.3-24.7)	19.4 (19.1-19.8)	19.6 (19.2-19.9)	19.6 (19.3-19.9)	23.9 (23.6-24.2)	37.7 (37.2-38.1)	<.001
Hepatitis B virus	4.4 (4.4-4.5)	4.9 (4.7-5.1)	4.8 (4.6-5.0)	4.4 (4.2-4.6)	4.1 (3.9-4.2)	4.1 (3.9-4.3)	<.001
Hepatitis C virus	31.6 (31.3-31.9)	35.2 (34.4-35.9)	33.6 (32.9-34.3)	31.9 (31.2-32.5)	30.3 (29.7-30.9)	27.9 (27.3-28.5)	<.001
Nonalcoholic fatty liver disease	19.8 (19.7-20.0)	18.6 (18.2-19.0)	19.9 (19.5-20.3)	21.4 (21.0-21.8)	21.4 (21.0-21.8)	18.0 (17.6-18.4)	.67
Hepatocellular carcinoma	4.5 (4.4-4.6)	4.7 (4.4-5.0)	4.7 (4.5-5.0)	4.5 (4.3-4.8)	4.4 (4.2-4.6)	4.0 (3.8-4.2)	<.001
Cirrhosis	53.0 (52.8-53.2)	51.2 (50.6-51.7)	51.8 (51.3-52.4)	52.0 (51.5-52.5)	53.0 (52.5-53.5)	56.4 (55.9-56.8)	<.001
Cirrhosis-related complications, No.							
0	14.7 (14.6-14.8)	14.8 (14.5-15.0)	14.8 (14.6-15.0)	14.6 (14.4-14.9)	14.8 (14.6-15.0)	14.5 (14.2-14.7)	.08
1	25.7 (25.5-25.8)	24.7 (24.4-25.1)	25.0 (24.7-25.3)	24.8 (24.5-25.1)	25.4 (25.1-25.6)	28.1 (27.8-28.3)	<.001
≥2	12.6 (12.5-12.8)	11.6 (11.3-12.0)	12.0 (11.7-12.4)	12.6 (12.3-12.9)	12.8 (12.5-13.2)	13.8 (13.5-14.2)	<.001
Coronary artery disease	14.5 (14.4-14.6)	13.5 (13.2-13.8)	13.8 (13.5-14.1)	13.9 (13.7-14.2)	14.7 (14.5-15.0)	16.0 (15.8-16.3)	<.001
Congestive heart failure	14.5 (14.4-14.6)	12.9 (12.7-13.2)	13.4 (13.2-13.7)	14.3 (14.0-14.5)	15.1 (14.8-15.3)	16.4 (16.2-16.7)	<.001
Chronic kidney disease	20.7 (20.5-20.8)	18.6 (18.3-19.0)	19.4 (19.0-19.8)	20.2 (19.8-20.6)	21.3 (21.0-21.7)	23.3 (22.9-23.6)	<.001
Diabetes	31.8 (31.6-31.9)	30.6 (30.2-30.9)	31.3 (30.9-31.6)	31.7 (31.4-32.1)	32.4 (32.0-32.7)	32.6 (32.3-33.0)	<.001
Dyslipidemia	20.8 (20.6-21.0)	18.0 (17.7-18.4)	19.1 (18.7-19.5)	20.6 (20.2-21.0)	22.2 (21.8-22.6)	23.5 (23.0-23.9)	<.001
Hypertension	46.5 (46.3-46.7)	48.3 (47.9-48.8)	49.6 (49.2-50.1)	50.6 (50.1-51.1)	48.7 (48.3-49.1)	36.6 (36.3-36.9)	<.001
Obesity	14.2 (14.0-14.3)	11.8 (11.5-12.1)	12.9 (12.6-13.2)	14.2 (13.9-14.5)	15.3 (14.9-15.6)	16.3 (15.9-16.6)	<.001
Pneumonia	14.2 (14.0-14.3)	14.0 (13.7-14.2)	14.2 (13.9-14.5)	14.5 (14.2-14.7)	14.0 (13.8-14.3)	14.1 (13.9-14.3)	.76
Stroke	3.5 (3.4-3.5)	3.3 (3.2-3.4)	3.3 (3.2-3.4)	3.5 (3.4-3.6)	3.5 (3.4-3.6)	3.6 (3.5-3.7)	<.001

### Trends in CLD-Related Hospitalizations

The annual rate of CLD-related hospitalizations per 100 000 hospitalizations increased from 3056 (95% CI, 3042-3069) in 2012 to 3757 (95% CI, 3742-3772) in 2016 (*P* for trend < .001) (Table 1). The total number of CLD-related hospitalizations increased by 20.8% (187 690 in 2012 to 226 714 in 2016). When stratified by age, while the proportion of hospitalizations for patients aged 45 to 64 years decreased (55.5% [95% CI, 55.1%-55.9%] in 2012 to 50.3% [95% CI, 49.9%-50.6%] in 2016; *P* for trend < .001), the proportion for those aged 65 years or older showed a considerable increase (27.4% [95% CI, 26.9%-27.9%] in 2012 to 32.2% [95% CI, 31.7%-32.6%] in 2016; *P* for trend < .001). Mean (SD) age increased (56.8 [14.2] years in 2012 to 57.8 [14.6] years in 2016; *P* for trend < .001). There were also increases in the proportion of hospitalizations for patients enrolled in Medicare (41.7% [95% CI, 41.1%-42.2%] to 43.6% [95% CI, 43.1%-44.1%]; *P* for trend < .001). The proportion enrolled in Medicaid also increased, while the proportion of uninsured (ie, self-pay) hospitalizations decreased (Table 1). Among CLD etiologies, the proportion of hospitalizations with HBV and HCV decreased (Table 1), while the proportion with alcoholic cirrhosis increased (19.4% [95% CI, 19.1%-19.8%] in 2012 to 37.7% [95% CI, 37.2%-38.1%] in 2016) (*P* for trend < .001 for all). The proportion of hospitalizations with NAFLD increased from 18.6% (95% CI, 18.2%-19.0%) in 2012 to 21.4% (95% CI, 21.0%-21.8%) in 2015; however, it decreased to 18.0% (95% CI, 17.6%-18.4%) in 2016. The proportion of hospitalizations with cirrhosis and 1 or more concurrent complication showed substantial increases (Table 1). The comorbidity burden among CLD-related hospitalizations showed a significant increase for all comorbid conditions analyzed.

### In-Hospital Mortality Among CLD-Related Hospitalizations

Among CLD-related hospitalizations, the crude in-hospital mortality rate remained stable from 7.4% (95% CI, 7.2%-7.6%) in 2012 to 7.3% (95% CI, 7.1%-7.4%) in 2016 (*P* for trend = .33) (Table 2), while the overall adjusted in-hospital mortality rate showed a decrease (odds ratio, 0.96; 95% CI, 0.95-0.96; *P* for trend < .001) (eTable 3 in the Supplement). The proportion of in-hospital deaths increased with increasing age (Table 2). Despite lower proportions of Asian/Pacific Islander individuals among the total cohort, those with Asian/Pacific Islander or African American background had higher rates of in-hospital death compared with individuals of other races (Table 2). Rates of in-hospital mortality were higher among CLD-related hospitalizations with Medicare compared with other insurance types (Table 2). Among etiologies, hospitalizations with HBV and HCV had relatively low in-hospital mortality rates and showed small increases (Table 2), while hospitalizations with alcoholic cirrhosis had the highest in-hospital mortality rate and showed the greatest increase (11.1% [95% CI, 10.7%-11.5%] in 2012 to 13.4% [95% CI, 13.1%-13.7%] in 2016, *P* for trend < .001). The overall rate of alcoholic cirrhosis was 11.9% (95% CI, 11.7%-12.0%). The in-hospital mortality rate among hospitalizations of patients with cirrhosis increased significantly, and an increasing number of concurrent cirrhosis-related complications was associated with increasing in-hospital mortality rates (Table 2). For example, the in-hospital mortality rate among patients with 2 or more cirrhosis-related complications was considerably higher than the rate among patients who had cirrhosis but no cirrhosis-related complications (13.1% [95% CI, 12.9%-13.3%] vs 4.8% [95% CI, 4.7%-4.9%]; *P* < .001). Presence of hepatocellular carcinoma was also associated with a high mortality rate (9.8% [95% CI, 9.5%-10.1%]).

**Table 2.** Trends in In-Hospital Mortality

Characteristic	Proportion with in-hospital mortality, weighted % (95% CI)						*P* value for trend
	Total	2012	2013	2014	2015	2016	
Overall	7.4 (7.4-7.5)	7.4 (7.2-7.6)	7.5 (7.3-7.6)	7.6 (7.4-7.8)	7.4 (7.3-7.6)	7.3 (7.1-7.4)	.33
Age, y							
<25	2.6 (2.3-2.8)	2.7 (2.1-3.3)	2.2 (1.8-2.8)	2.5 (2.0-3.1)	2.6 (2.1-3.3)	2.8 (2.3-3.4)	.43
25-44	3.7 (3.5-4.0)	3.6 (3.4-3.9)	3.9 (3.6-4.1)	3.8 (3.6-4.1)	3.5 (3.3-3.8)	3.7 (3.5-4.0)	.77
45-64	6.5 (6.4-6.5)	6.4 (6.2-6.6)	6.5 (6.3-6.7)	6.5 (6.4-6.7)	6.5 (6.3-6.7)	6.4 (6.2-6.6)	.62
≥65	11.4 (11.3-11.5)	11.7 (11.4-12.0)	11.6 (11.3-11.9)	11.9 (11.6-12.2)	11.3 (11.0-11.6)	10.7 (10.5-11.0)	<.001
Sex							
Male	7.4 (7.4-7.5)	7.4 (7.2-7.6)	7.5 (7.3-7.7)	7.6 (7.4-7.8)	7.4 (7.2-7.6)	7.3 (7.1-7.5)	.39
Female	7.4 (7.3-7.5)	7.4 (7.2-7.6)	7.5 (7.3-7.7)	7.6 (7.4-7.8)	7.5 (7.3-7.7)	7.3 (7.1-7.5)	.47
Race							
White	7.5 (7.4-7.6)	7.5 (7.3-7.7)	7.5 (7.3-7.7)	7.7 (7.5-7.9)	7.5 (7.3-7.7)	7.3 (7.1-7.5)	.23
African American	7.7 (7.5-7.8)	7.4 (7.0-7.8)	7.5 (7.2-7.9)	7.8 (7.4-8.2)	8.0 (7.6-8.4)	7.7 (7.4-8.0)	.09
Hispanic	6.3 (6.1-6.4)	6.4 (6.1-6.8)	6.5 (6.2-6.9)	6.4 (6.1-6.8)	6.0 (5.7-6.4)	6.0 (5.7-6.3)	.02
Asian/Pacific Islander	8.8 (8.4-9.2)	8.7 (7.8-9.6)	9.3 (8.4-10.2)	9.0 (8.2-9.9)	8.7 (7.9-9.5)	8.3 (7.6-9.1)	.25
Insurance							
Medicare	9.1 (9.0-9.2)	9.1 (8.8-9.3)	9.2 (8.9-9.4)	9.4 (9.1-9.6)	9.1 (8.9-9.4)	8.8 (8.6-9.0)	.12
Medicaid	5.5 (5.4-5.6)	5.5 (5.3-5.8)	5.8 (5.5-6.1)	5.6 (5.3-5.8)	5.3 (5.0-5.5)	5.4 (5.2-5.6)	.05
Private, including health maintenance organization	6.8 (6.7-6.9)	6.9 (6.6-7.2)	6.6 (6.4-6.9)	6.8 (6.5-7.1)	6.9 (6.6-7.1)	6.7 (6.4-7.0)	.77
Self-pay	6.0 (5.8-6.2)	5.9 (5.5-6.3)	6.0 (5.6-6.5)	6.4 (5.9-6.9)	5.9 (5.5-6.4)	5.6 (5.2-6.1)	.46
Alcoholic cirrhosis	11.9 (11.7-12.0)	11.1 (10.7-11.5)	10.7 (10.3-11.0)	10.7 (10.4-11.1)	11.8 (11.4-12.1)	13.4 (13.1-13.7)	<.001
Hepatitis B virus	4.4 (4.2-4.6)	4.3 (3.8-4.7)	4.2 (3.8-4.7)	4.5 (4.1-5.0)	4.4 (4.0-4.9)	4.4 (4.0-4.9)	.43
Hepatitis C virus	4.1 (4.1-4.2)	3.9 (3.7-4.1)	4.2 (4.0-4.4)	4.2 (4.0-4.4)	4.3 (4.2-4.5)	4.0 (3.8-4.1)	.44
Nonalcoholic fatty liver disease	4.1 (4.0-4.3)	4.8 (4.5-5.0)	4.6 (4.4-4.9)	4.7 (4.5-4.9)	4.0 (3.8-4.2)	2.7 (2.5-2.9)	<.001
Hepatocellular carcinoma	9.8 (9.5-10.1)	9.7 (9.0-10.4)	9.4 (8.6-10.1)	9.9 (9.2-10.6)	10.1 (9.4-10.8)	9.8 (9.2-10.5)	.37
Cirrhosis	9.1 (9.0-9.2)	8.3 (8.1-8.5)	8.1 (7.9-8.4)	8.3 (8.1-8.5)	9.0 (8.8-9.2)	11.2 (10.9-11.4)	<.001
Cirrhosis-related complications							
0	4.8 (4.7-4.9)	5.0 (4.7-5.3)	5.3 (5.0-5.5)	5.2 (4.9-5.5)	4.6 (4.4-4.9)	4.1 (3.9-4.3)	<.001
1	9.6 (9.4-9.7)	8.0 (7.7-8.2)	7.6 (7.3-7.9)	7.9 (7.6-8.1)	9.6 (9.3-9.8)	13.6 (13.2-13.9)	<.001
≥2	13.1 (12.9-13.3)	13.2 (12.7-13.7)	12.8 (12.3-13.3)	12.8 (12.3-13.3)	12.9 (12.5-13.4)	13.7 (13.2-14.1)	.09
Coronary artery disease	9.4 (9.2-9.6)	9.2 (8.8-9.6)	9.4 (9.0-9.8)	9.7 (9.3-10.1)	9.2 (8.9-9.6)	9.4 (9.1-9.8)	.71
Congestive heart failure	12.8 (12.6-13.0)	12.8 (12.4-13.3)	13.5 (13.0-13.9)	13.3 (12.8-13.7)	12.7 (12.3-13.1)	12.1 (11.8-12.5)	<.001
Chronic kidney disease	10.2 (10.1-10.4)	10.3 (10.0-10.7)	10.5 (10.2-10.9)	10.5 (10.2-10.9)	10.3 (10.0-10.6)	9.7 (9.5-10.0)	.002
Diabetes	6.6 (6.5-6.7)	6.6 (6.4-6.8)	6.6 (6.3-6.8)	6.6 (6.4-6.9)	6.6 (6.4-6.8)	6.5 (6.3-6.7)	.31
Dyslipidemia	6.5 (6.3-6.6)	6.3 (6.0-6.5)	6.3 (6.0-6.6)	6.7 (6.4-7.0)	6.5 (6.2-6.7)	6.5 (6.3-6.8)	.10
Hypertension	6.6 (6.5-6.7)	6.8 (6.6-7.0)	6.8 (6.6-7.0)	7.0 (6.8-7.2)	6.7 (6.6-6.9)	5.6 (5.4-5.8)	<.001
Obesity	6.2 (6.0-6.3)	6.3 (5.9-6.6)	6.1 (5.7-6.4)	6.3 (6.0-6.7)	6.2 (5.9-6.5)	6.0 (5.8-6.3)	.46
Pneumonia	15.2 (15.0-15.4)	15.1 (14.6-15.6)	15.3 (14.8-15.8)	15.7 (15.2-16.2)	15.2 (14.7-15.6)	14.8 (14.4-15.3)	.33
Stroke	14.2 (13.8-14.6)	13.4 (12.6-14.3)	13.6 (12.7-14.5)	14.8 (13.9-15.7)	14.4 (13.6-15.3)	14.5 (13.7-15.2)	.04

### Hospitalization Costs Among CLD-Related Hospitalizations

**Figure 1.**
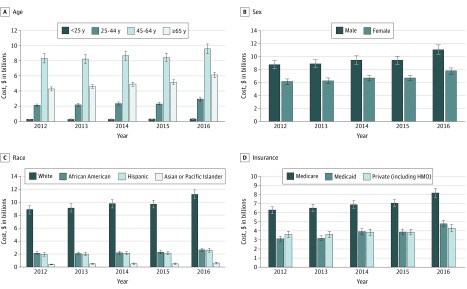
Trends in Total Hospitalization Costs Stratified by Demographic Characteristics Costs are shown in 2016 US dollars. Error bars represent 95% CIs; HMO, health maintenance organization.

The mean cost per CLD-related hospitalization was $16 271 (95% CI, $16 069-$16 473), while the total national estimated cost for CLD-related hospitalizations over the study period was $81.1 billion (95% CI, $79.4 billion to $82.7 billion). The adjusted mean cost per CLD-related hospitalization increased by 0.62% (95% CI, 0.07%-1.18%; *P* for trend = .03) over the study period (eTable 3 in the Supplement). From 2012 to 2016, mean hospitalization costs increased from $15 611 (95% CI, $13 870-$17 353) to $17 478 (95% CI, $15 653-$19 304) (*P* for trend < .001), and total hospitalization costs increased from $14.9 billion (95% CI, $13.9 billion to $15.9 billion) in 2012 to $18.8 billion (95% CI, $17.6 billion to $20.0 billion) in 2016 (Table 3). While mean and total hospitalization costs increased for all age groups (Table 3 and Figure 1A), total costs were highest among hospitalized patients aged 45 to 64 years (Figure 1A). Mean hospitalization costs increased significantly among men and women; however, men had a higher total cost burden ($47.6 billion [95% CI, $46.5 billion to $48.6 billion] vs $33.5 billion [95% CI, $32.8 billion to $34.2 billion]) (Figure 1B). While African American individuals had the most significant increase in mean hospitalization costs, mean costs were highest among Asian/Pacific Islander individuals (Table 3), and white patients had the highest total economic burden ($48.5 billion [95% CI, $47.5 billion to $49.6 billion]) (Figure 1C).

**Table 3.** Trends in Mean Hospitalization Costs

Characteristic	Costs, weighted mean (95% CI), 2016 US dollars					*P* value for trend
	2012	2013	2014	2015	2016	
Total costs (95% CI), 2016 US dollars, billions	14.9 (13.9-15.9)	15.2 (14.2-16.2)	16.1 (15.0-17.1)	16.1 (15.1-17.1)	18.8 (17.6-20.0)	
Overall	16 168 (15 667-16 670)	16 094 (15 618-16 571)	16 332 (15 821-16 843)	15 763 (15 324-16 203)	16 919 (16 417-17 422)	<.001
Age, y						
<25	15 611 (13 870-17 353)	15 731 (14 043-17 420)	16 958 (15 234-18 681)	16 823 (15 434-18 212)	17 478 (15 653-19 304)	<.001
25-44	14 607 (13 969-15 245)	14 766 (14 136-15 397)	15 098 (14 482-15 714)	14 239 (13 702-14 777)	16 038 (15 371-16 704)	<.001
45-64	16 211 (15 656-16 765)	16 009 (15 501-16 516)	16 389 (15 822-16 955)	15 842 (15 365-16 319)	17 071 (16 500-17 642)	<.001
≥65	17 013 (16 543-17 483)	16 996 (16 509-17 483)	16 855 (16 398-17 313)	16 361 (15 944-16 779)	17 100 (16 674-17 527)	.01
Sex						
Male	16 424 (15 883-16 966)	16 458 (15 938-16 979)	16 738 (16 184-17 291)	16 180 (15 710-16 650)	17 426 (16 870-17 982)	<.001
Female	15 818 (15 335-16 302)	15 601 (15 140-16 062)	15 789 (15 299-16 278)	15 212 (14 785-15 639)	16 247 (15 780-16 713)	<.001
Race						
White	15 540 (15 031-16 050)	15 475 (14 998-15 952)	15 710 (15 214-16 205)	15 079 (14 672-15 487)	15 994 (15 531-16 457)	<.001
African American	16 615 (16 046-17 185)	16 494 (15 905-17 083)	16 923 (16 340-17 510)	16 873 (16 311-17 435)	18 036 (17 370-18 703)	<.001
Hispanic	16 391 (15 275-17 507)	16 516 (15 387-17 646)	16 848 (15 848-17 849)	15 773 (14 899-16 647)	17 434 (16 427-18 442)	<.001
Asian/Pacific Islander	21 278 (19 163-23 392)	22 312 (20 711-23 913)	22 383 (20 374-24 391)	20 262 (18 984-21 540)	22 302 (20 516-24 088)	.01
Insurance						
Medicare	16 435 (15 953-16 916)	16 384 (15 912-16 856)	16 391 (15 911-16 870)	15 976 (15 562-16 391)	16 945 (16 504-17 387)	<.001
Medicaid	15 660 (15 052-16 268)	15 748 (15 215-16 281)	16 215 (15 673-16 757)	15 335 (14 868-15 803)	17 229 (16 631-17 826)	<.001
Private, including health maintenance organization	17 832 (16 982-18 681)	17 558 (16 760-18 355)	17 615 (16 758-18 473)	16 934 (16 202-17 667)	17 726 (16 919-18 533)	
Self-pay	12 728 (12 250-13 207)	12 690 (12 215-13 165)	12 944 (12 487-13 400)	12 277 (11 821-12 732)	13 160 (12 442-13 878)	
Alcoholic cirrhosis	17 787 (17 140-18 434)	17 615 (16 963-18 266)	17 677 (16 977-18 376)	17 453 (16 877-18 028)	20 282 (19 560-21 004)	<.001
Hepatitis B virus	15 203 (14 429-15 977)	15 454 (14 659-16 248)	16 029 (15 112-16 947)	15 533 (14 749-16 318)	16 970 (16 069-17 871)	<.001
Hepatitis C virus	13 991 (13 502-14 479)	14 164 (13 669-14 659)	14 290 (13 810-14 770)	14 197 (13 758-14 636)	15 085 (14 602-15 568)	<.001
Nonalcoholic fatty liver disease	13 566 (13 119-14 013)	13 346 (12 924-13 769)	13 456 (13 021-13 891)	13 203 (12 847-13 560)	13 769 (13 387-14 151)	<.001
Hepatocellular carcinoma	18 512 (17 162-19 863)	18 111 (16 528-19 694)	17 861 (16 579-19 143)	18 049 (16 718-19 380)	18 952 (17 563-20 341)	.14
Cirrhosis	16 285 (15 663-16 908)	16 190 (15 573-16 806)	16 309 (15 641-16 976)	16 298 (15 726-16 870)	19 185 (18 485-19 885)	<.001
Cirrhosis-related complications						
0	13 313 (12 868-13 759)	13 402 (12 950-13 855)	13 390 (12 950-13 829)	13 165 (12 803-13 528)	13 616 (13 246-13 985)	<.001
1	15 303 (14 775-15 831)	15 170 (14 652-15 689)	15 169 (14 673-15 666)	15 747 (15 280-16 213)	20 041 (19 415-20 667)	<.001
≥2	22 185 (20 858-23 512)	21 759 (20 530-22 988)	21 958 (20 418-23 498)	21 027 (19 821-22 233)	23 279 (21 718-24 841)	.20
Coronary artery disease	17 096 (16 531-17 660)	16 699 (16 150-17 249)	17 297 (16 714-17 880)	16 627 (16 195-17 059)	17 712 (17 194-18 230)	<.001
Congestive heart failure	23 012 (22 073-23 951)	21 906 (21 118-22 695)	22 530 (21 704-23 356)	21 292 (20 600-21 984)	22 489 (21 715-23 263)	.20
Chronic kidney disease	19 803 (18 940-20 667)	19 228 (18 526-19 929)	19 517 (18 742-20 291)	18 562 (17 960-19 164)	20 049 (19 274-20 823)	.01
Diabetes	15 800 (15 321-16 279)	15 464 (15 028-15 900)	15 553 (15 093-16 014)	15 373 (14 958-15 788)	16 213 (15 769-16 657)	<.001
Dyslipidemia	15 094 (14 636-15 553)	14 994 (14 614-15 374)	15 373 (14 949-15 797)	14 962 (14 618-15 306)	15 787 (15 386-16 186)	<.001
Hypertension	15 930 (15 448-16 413)	15 529 (15 107-15 951)	15 843 (15 394-16 292)	15 209 (14 839-15 579)	14 913 (14 527-15 299)	.56
Obesity	17 692 (17 096-18 287)	17 318 (16 750-17 887)	17 471 (16 909-18 032)	17 200 (16 685-17 714)	18 162 (17 591-18 734)	.01
Pneumonia	27 959 (26 718-29 200)	27 066 (25 973-28 160)	27 815 (26 694-28 936)	25 710 (24 768-26 653)	28 360 (27 217-29 503)	.17
Stroke	25 569 (24 115-27 024)	24 944 (23 507-26 381)	25 885 (24 499-27 272)	24 571 (23 348-25 794)	28 509 (26 978-30 039)	<.001

**Figure 2.**
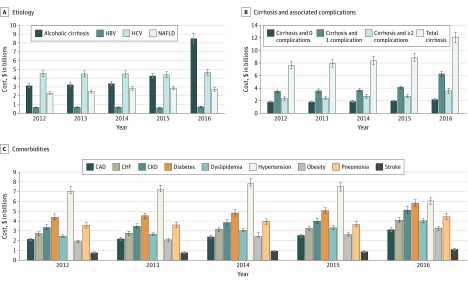
Trends in Total Hospitalization Costs Stratified by Clinical Characteristics Costs are shown in 2016 US dollars. CAD indicates coronary artery disease; CHF, congestive heart failure; CKD, chronic kidney disease; HBV, hepatitis B virus; HCV, hepatitis C virus; NAFLD, nonalcoholic fatty liver disease; and error bars, 95% CIs.

Compared with other insurance types, hospitalizations of patients with private insurance had higher mean costs (Table 3), hospitalizations of those enrolled in Medicare had a higher total burden (Figure 1D), and mean costs for hospitalizations of patients enrolled in Medicaid experienced the greatest increase ($15 660 [95% CI, $15 052-$16 268] in 2012 to $17 229 [95% CI, $16 631-$17 826] in 2016; *P* for trend < .001). Cost burden increased across all etiologies, with a higher total cost burden among hospitalizations with alcoholic cirrhosis ($22.7 billion [95% CI, $22.1 billion to $23.2 billion]) or hepatitis C virus ($22.6 billion [95% CI, $22.1 billion to $23.2 billion]) (Table 3 and Figure 2A). Mean costs per hospitalization were higher among patients with alcoholic cirrhosis and HBV compared with the other etiologies. Hospitalizations for patients with HCV had the highest total costs from 2012 to 2015, and those with alcoholic cirrhosis had the highest total costs in 2016 (Figure 2A). Hospitalization costs increased among patients with cirrhosis, with a higher burden among patients with concurrent cirrhosis-related complications (Table 3 and Figure 2B). While CLD-related hospitalizations with concurrent congestive heart failure, pneumonia, or stroke had relatively higher mean costs, hospitalizations with concurrent diabetes and hypertension had a higher total cost burden compared with other comorbidities (Figure 2C).

## Discussion

Among a large, nationally representative sample of hospitalized US adults from 2012 to 2016, there was a 20.8% increase in the number of CLD-related hospitalizations, resulting in a 26.2% increase in the total estimated hospitalization costs. In 2016, CLD-related hospitalizations alone accounted for $18.8 billion of the economic burden.

The observed increases in the clinical and economic burden of CLD among hospitalized adults in the US is a major public health concern. Improvements in the management of viral hepatitis, specifically the introduction of direct-acting antivirals for HCV treatment,^29^ have led to significantly reduced morbidity and mortality associated with HBV and HCV.^30,31,32,33,34,35,36^ However, existing studies^4,11^ highlight the growing epidemic of NAFLD in the US. Studies^2,5,6^ have also shown the rising prevalence of ALD and increasing disease severity among patients with ALD, with consistent trends across different nationally representative databases. In this study, while the proportion of patients with alcoholic cirrhosis and NAFLD showed an increasing trend, the highest mortality burden was among hospitalizations with alcoholic cirrhosis, and the major cost burden was distributed between hospitalizations of patients with alcoholic cirrhosis and HCV. Even with decreasing HCV-associated morbidity and mortality, the high HCV cost burden may be due to aging patients with HCV cirrhosis and continued suboptimal access to HCV treatment.^37,38^

These epidemiological trends likely reflect the lack of early diagnosis, of ALD and NAFLD in particular, and the paucity of effective therapies to treat and prevent disease progression. The findings of the current study raise concern regarding the vast burden of CLD on the US health care system, and the impending wave of patients with ALD and NAFLD will further stress the health care system unless precautionary actions are taken. There is an urgent need for greater awareness among patients and clinicians about the consequences of unhealthy alcohol use, the need for consistent screening for alcohol use disorder, and timely implementation of prevention and intervention strategies aimed specifically at addressing alcohol use disorders.

In this study, presence of cirrhosis and cirrhosis-related complications resulted in a higher mortality rate and cost burden on the health care system. Tapper et al^39^ applied a quality improvement intervention on an inpatient liver unit that resulted in a 40% reduction in 30-day readmissions among patients with cirrhosis, which was likely driven by a decrease in readmissions for hepatic encephalopathy. Kanwal et al^40^ demonstrated that while early clinic follow-up following hospital discharge led to an increase in readmissions, this ultimately translated into reduced overall mortality. Similarly, other studies have proposed quality improvement tools and shown that prevention and early management of cirrhosis are cost-effective.^41,42,43,44,45,46^ However, owing to the lack of nationally representative data to guide the optimal allocation of resources and preventive efforts, there is a lag in the application and use of these evidence-based protocols by clinicians.

With advancements in medical care, there is a shift in the burden toward an older population. This is reflected in the current study, which observed increases in the mean age of hospitalized patients with CLD and found that 42.7% of hospitalizations were for patients enrolled in Medicare. This is particularly concerning given that older populations have higher risk of comorbidities. A recent study by Younossi et al^4^ showed that increases in the prevalence of NAFLD were associated with increasing rates of obesity and type 2 diabetes.^47,48,49^ This study demonstrated similar observations showing concurrent increases in the comorbidity burden, albeit among a hospitalized cohort. As with prior studies, the presence of these comorbidities was associated with worse prognosis among this CLD cohort.^6,50,51^

The proportion of CLD-related hospitalizations for patients enrolled in Medicare increased significantly over the study period, and hospitalizations for those with Medicare experienced the highest in-hospital mortality rate as well as the greatest total economic burden. Asrani et al^14^ found a similar trend among hospitalizations covered by Medicare (increase from 31.8% to 41.5%). These observations, along with the results of the current study, draw attention to the imminent surge in the burden of older patients with CLD and call attention to the need for greater research aimed at preventive care and novel quality-driven health care delivery so that we may adequately address the future needs of patients with CLD in a cost-effective and cost-efficient manner.

### Limitations

As with many studies using large claims databases, these findings should be interpreted within the context of certain limitations.^52^ The NIS records data per hospitalization, and not per individual patient, without linkage or follow-up data. Owing to the cross-sectional nature of the database, longitudinal analysis is not possible, and no causal inferences can be drawn. Thus, there may be repeat hospitalizations of patients with CLD among this study cohort. Nevertheless, given that the main objective of this study was to characterize the overall clinical and economic burden of CLD hospitalizations in the US, these results still provide critical data to address this important public health concern. This study focused specifically on alcoholic cirrhosis to minimize error in the estimates given the limitations inherent in the use of *ICD-9-CM* coding-based algorithms to identify and isolate ALD as a whole. However, because this study focused specifically on alcoholic cirrhosis, it may not capture the true burden of ALD (eg, the burden among patients with alcoholic hepatitis who may be hospitalized but do not have cirrhosis). A considerable proportion of hospitalizations did not have an identifiable underlying etiology. Hospitalizations in this category related to CLD may be attributable to other chronic diseases (eg, cholestatic liver diseases). The changes in coding and data structure of the NIS in 2015 may have resulted in misclassification bias,^21^ and the *P* value for trend for some characteristics may not be as informative owing to these changes. Chronic liver disease with mention of alcohol is more thoroughly defined in *ICD-10-CM* (K70.xx) compared with the *ICD-9-CM*; however, NAFLD *ICD* codes still lack specificity in both coding systems. This change may have contributed to the apparent increased burden of alcoholic cirrhosis and decreased burden of NAFLD in 2016. The loss of Elixhauser comorbidity software within the NIS for the fourth quarter of 2015 and for 2016 may have led to underestimation of the burden of certain comorbidities (eg, hypertension). We tried to circumvent these limitations through a comprehensive review of *ICD* codes to ensure that our definitions using *ICD-10-CM* codes are congruent with the *ICD-9-CM* codes previously used.

## Conclusions

While this study is unable to account for outpatient outcomes, including mortality following hospitalization and long-term costs associated with chronic care, inpatient burden is a common and relevant metric used to estimate the health care burden for chronic conditions. Similar to other chronic diseases, a large proportion of CLD-related health care burden is reflected in inpatient mortality and resource utilization. In addition, severe decompensation often leads to hospitalization, which has been linked to early mortality among patients with other chronic conditions.^46,53,54,55^ Thus, despite inherent limitations and potential underestimation of the true burden of CLD, this study provides a comprehensive view of trends in CLD-related inpatient resource utilization in the US as a stepping stone to guide health care policy and more efficient allocation of resources to address the looming burden of an aging CLD population with higher morbidity and mortality.

## Notes

Supplement. **eFigure.** Flow Diagram for the Identification of CLD-Related Hospitalizations and Categorization Into the Etiologies of Interest for Trends Analysis **eTable 1.** List of ICD Codes Used in CLD Definition **eTable 2.** List of ICD Codes Used for Other Definitions **eTable 3.** Regression Models for Trends in In-Hospital Mortality and Mean Hospitalization Costs Among CLD-Related Hospitalizations
