# Supplementary material for: Trends in the Burden of Chronic Liver Disease Among Hospitalized US Adults
Source: JAMA Netw Open. 2020 Apr 2;3(4):e201997. doi: 10.1001/jamanetworkopen.2020.1997 (PMC7118516; doi:10.1001/jamanetworkopen.2020.1997)
Supplement: Supplement. — eFigure. Flow Diagram for the Identification of CLD-Related Hospitalizations and Categorization Into the Etiologies of Interest for Trends Analysis eTable 1. List of ICD Codes Used in CLD Definition eTable 2. List of ICD Codes Used for Other Definitions eTable 3. Regression Models for Trends in In-Hospital Mortality and Mean Hospitalization Costs Among CLD-Related Hospitalizations [file jamanetwopen-3-e201997-s001.pdf]

# Supplementary Online Content

Hirode G, Saab S, Wong RJ. Trends in the burden of chronic liver disease among hospitalized US adults. *JAMA Netw Open*. 2020;3(4):e201997. doi:10.1001/jamanetworkopen.2020.1997

**eFigure.** Flow Diagram for the Identification of CLD-Related Hospitalizations and Categorization Into the Etiologies of Interest for Trends Analysis

**eTable 1.** List of ICD Codes Used in CLD Definition

**eTable 2.** List of ICD Codes Used for Other Definitions

**eTable 3.** Regression Models for Trends in In-Hospital Mortality and Mean Hospitalization Costs Among CLD-Related Hospitalizations

This supplementary material has been provided by the authors to give readers additional information about their work.

© 2020 Hirode G et al. *JAMA Network Open.*

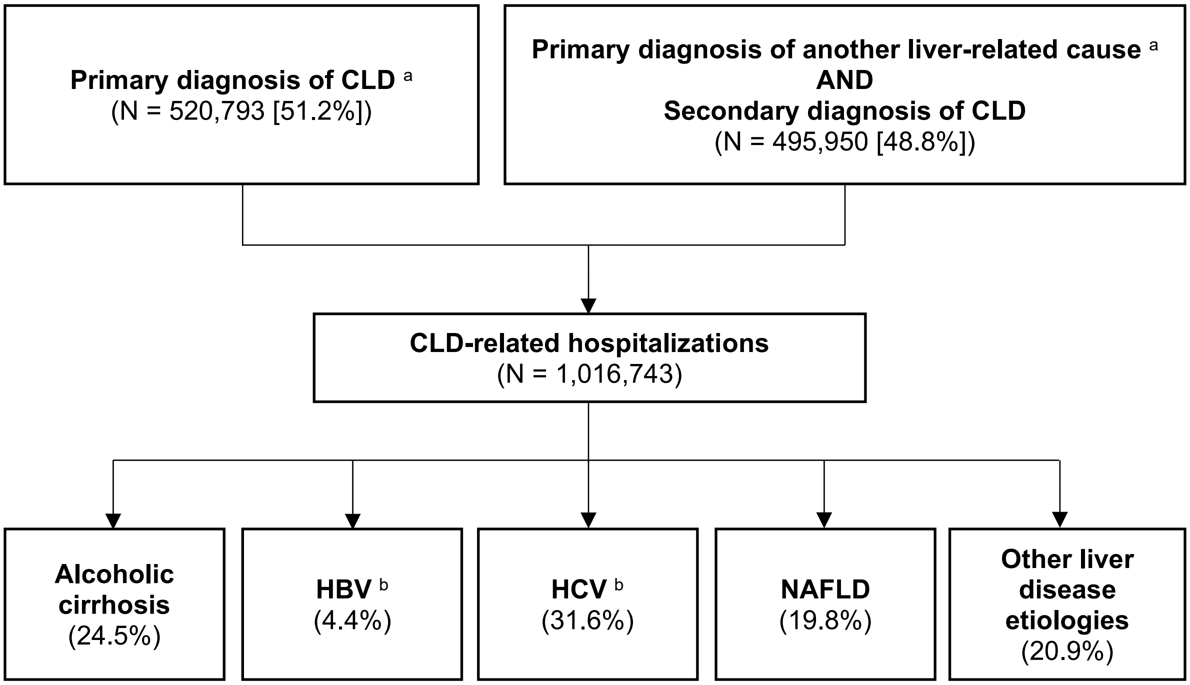


**eFigure. Flow diagram for the identification of CLD-related hospitalizations and categorization into the etiologies of interest for trends analysis.**

[a] See eTable 1 and eTable 2 for ICD codes.

[b] 12,910 hospitalizations were co-infected with HBV and HCV, and were included in both sub-cohorts.

© 2020 Hirode G et al. *JAMA Network Open.*

**eTable 1.** List of ICD codes used in CLD definition.

| | ICD-9-CM | ICD-10-CM |
|---|---|---|
| **Definition of chronic liver disease-related diagnosis** | | |
| Acute and subacute necrosis of liver | 570 | K72.0x, K76.2 |
| Chronic liver disease and cirrhosis | 571.xx | K70.xx, K72.1x, K73.x, K74.xx, K75.4, K75.8x, K76.0, K76.89, K76.9 |
| Liver abscess and sequelae of chronic liver disease | 572.x | K72.9x, K75.0-1, K76.6-7 |
| Malignant neoplasm of liver and intrahepatic bile duct | 155.x | C22.0-2, C22.7-9 |
| Other disorders of the liver | 573.x | K71.0-1x, K71.3-9, K72, K75, K75.3, K75.9, K76.1, K76.8x, K76.9, K77 |
| Viral hepatitis | 070.xx, V02.6x | B15.x - B19.xx |
| **Diagnosis of another liver-related cause** | | |
| Acute kidney injury | 593.9 | N17.9, N28.9 |
| Ascites | 789.5x | K70.11, K70.31, K71.51, R18.x |
| Cellulitis | 681.xx, 682.x | K12.2, L03.01x, L03.03x, L03.11x, L03.211, L03.213, L03.221, L03.31x, L03.81x, L03.90 |
| Cholangitis | 576.1 | K83.0x |
| Encephalopathy | 348.3x, 572.2 | G93.4x, K72.xx |
| Esophageal varices | 456.0-2x | I85.xx |
| Gastrointestinal hemorrhage | 578.x | K92.0-2 |
| Hepatorenal syndrome | 572.4 | K76.7 |
| Hydrothorax | 511.89 | J90, J94.8 |
| Hyponatremia | 276.1 | E87.1 |
| Infection (including sepsis, pneumonia) | 008.xx, 009.x, 038.xx, 041.xx, 480.x - 487.x | A04.xx, A08.xx, A09, A40.x, A41.x, A48.1, A48.3, A48.8, A49.0x, A49.1, A49.8-9, B25.0, B44.0, B44.1, B49, B95.xx, B96.xx, J10.0x, J11.0x, J12.xx, J13, J14, J15.xx, J16.x, J17, J18.0-1, J18.9 |
| Jaundice | 782.4 | R17 |
| Liver failure | 570 | K70.4x, K72.xx |
| Peritonitis | 567.xx | K65.x, K67, K68.12, K68.19, K68.9 |
| Portal hypertension | 572.3 | K76.6 |
| Portal vein thrombosis or deep vein thrombosis | 451.xx - 454.x | I80.1x - I80.9, I81, I82.0 - I82.70x, I82.72x, I82.Axx - I82.Cxx, I82.89x, I82.9x |
| Renal failure | 585.x, 586, 794.4 | N18.x, N19, R94.4 |
| Sepsis | 038.xx, 785.52, 995.91, 995.92 | A40.x, A41.x, R65.2x |
| Volume overload or dehydration | 276.51, 276.6x, 782.3 | E86.0, E87.7x, R60.x |

© 2020 Hirode G et al. *JAMA Network Open.*

**eTable 2.** List of ICD codes used for other definitions.

| | ICD-9-CM | ICD-10-CM |
|---|---|---|
| Autoimmune hepatitis | 571.42 | K75.4 |
| Alcohol use disorder | 291.xx, 303.xx, 305.0x, 357.5, 425.5, 571.0-3, V11.3 | F10.xxx, G62.1, I42.6, K70.xx |
| Budd-Chiari syndrome | 453.0 | I82.0 |
| Coronary artery disease | 414.xx | I25.xxx |
| Congestive heart failure | 398.91, 402.01, 402.11, 402.91, 404.01, 404.03, 404.11, 404.13, 404.91, 404.93, 428.xx, 429.4, 997.1 | I09.81, I11.0, I13.0, I13.2, I50.xxx, I97.0, I97.110, I97.130, I97.190, I97.710, I97.790, I97.88, I97.89 |
| Chronic liver disease with mention of alcohol | 571.0-3 | K70.xx |
| Chronic passive congestion of liver | 573.0 | K76.1 |
| Cirrhosis | 571.2, 571.5, 567.23, 572.2, 572.4, 456.0-2x, 789.5x | I85.xx, K65.2, K70.11, K70.2-4x, K71.51, K71.7, K72.xx, K74.0-2, K74.6x, K76.7, R18.x |
| Chronic kidney disease | 585.x, 586, 794.4 | N18.x, N19, R94.4 |
| Clonorchiasis | 121.1 | B66.1 |
| Diabetes | 250.xx | E10.xxxx - E11.xxxx |
| Disorders of porphyrin and bilirubin metabolism | 277.1, 277.4 | E80.xx |
| Dyslipidemia | 272.0-4 | E78.0x-5 |
| Echinococcus of liver | 122.0, 122.5, 122.8 | B67.0, B67.5, B67.8 |
| Fascioliasis | 121.3 | B66.3 |
| Gaucher disease | 272.7 | E75.21-22, E75.249, E77.0-1 |
| Hemachromatosis | 275.0x | E83.1xx |
| Hepatitis A virus | 070.0, 070.1 | B15.x |
| Hepatitis B virus | 070.2x, 070.3x, V02.61 | B16.x, B18.0-1, B19.1x |
| Hepatitis C virus | 070.41, 070.44, 070.51, 070.54, 070.7x, V02.62 | B17.1x, B18.2, B19.2x |
| Hepatic encephalopathy | 572.2 | K72.xx |
| Hepatocellular carcinoma | 155.0 | C22.0, C22.7, C22.8 |
| Hypertension | 401.x | I10, I16.xx |
| Lysosomal acid lipase deficiency and other lipoid disorders | 272.8-9 | E78.8x, E78.9 |
| Obesity | 278.00, 278.01, 278.03, V85.3x, V85.4x | E66.0x-2, E66.8-9, Z68.3x-4x |
| Opisthorchiasis | 121.0 | B66.0 |
| Other amyloidosis | 277.3x | E85.xx |
| Other deficiencies of circulating enzymes | 277.6 | D84.1 |

© 2020 Hirode G et al. *JAMA Network Open.*

| | | |
|---|---|---|
| Other specified/unspecified disorders of the liver | 573.1-3, 573.8-9 | K71.xx, K74.4, K75.0-4, K76.2-5 |
| Other viral hepatitis | 070.4, 070.42, 070.43, 070.49, 070.5, 070.52, 070.53, 070.59, 070.6, 070.9, 571.4, 571.40, 571.41, 571.49, V02.6, V02.60, V02.69 | B17.0, B17.2, B17.8-9, B18.8-9, B19.0, B19.9, K71.6, K73.x |
| Pneumonia | 480.x - 486, 487.0 | A37.01, A37.11, A37.81, A37.91, A48.1, B25.0, B44.0, B44.1, J10.0x, J11.0x, J12.xx, J13, J14, J15.xx, J16.x, J17, J18.0-1, J18.9 |
| Primary biliary cholangitis | 571.6 | K74.3, K74.5 |
| Primary sclerosing cholangitis | 576.1 | K83.01 |
| Stroke | 430 - 438.xx | G45.x, G46.x, I60.xx - I69.xx |
| Syphilis of the liver | 095.3 | A52.74 |
| Wilson's disease | 275.1 | E83.0x |

© 2020 Hirode G et al. *JAMA Network Open.*

**eTable 3.** Regression models for trends in in-hospital mortality and mean hospitalization costs among CLD-related hospitalizations[a].

| | **In-hospital mortality** | | **Mean hospitalization costs** | |
|---|---|---|---|---|
| | **OR (95% CI)** | **P** | **% change (95% CI)** | **P** |
| **Year** | 0.96 (0.95-0.96) | <0.001 | 0.62% (0.07% - 1.18%) | 0.028 |

[a] Adjusted for demographic (age, sex, race and insurance status), etiologies (presence of hepatitis B virus, hepatitis C virus, alcoholic cirrhosis, nonalchoholic fatty liver disease), presence of hepatocellular carcinoma, presence of cirrhosis and complications (0, 1, ≥2), and comorbidities (coronary artery disease, congestive heart failure, chronic kidney disease, diabetes, dyslipidemia, hypertension, obesity, pneumonia, stroke).

© 2020 Hirode G et al. *JAMA Network Open.*
